# Pathology and host-pathogen interactions in a golden Syrian hamster model of Nipah virus infection

**DOI:** 10.3389/fvets.2025.1518358

**Published:** 2025-03-07

**Authors:** Inés Ruedas-Torres, Stephen Findlay-Wilson, Emma Kennedy, Stuart Dowall, Francisco Javier Salguero

**Affiliations:** United Kingdom Health Security Agency (UKHSA), Porton Down, Salisbury, United Kingdom

**Keywords:** Nipah virus, golden Syrian hamster, immunopathogenesis, histopathology, immunohistochemistry, henipaviruses

## Abstract

Nipah virus (NiV) is recognized as one of the key pathogens with pandemic potential. We have recently established a NiV hamster model, which reproduces a highly similar disease to that observed in human cases, including respiratory and neurological signs and lesions. The aims of this study were to describe the microscopic lesions observed in the golden Syrian hamster model after intranasal (IN) and intraperitoneal (IP) inoculation with different doses of the Malaysian strain of NiV; to describe in depth the cell composition of the pulmonary and the brain lesions and the expression of proinflammatory cytokines *in-situ* using a combination of histopathological techniques including immunohistochemistry (IHC) and *in-situ* hybridisation (ISH) via RNAscope technique. We also developed a multiplex IHC which will allow us to study the interaction of the virus with cell populations in the lung and brain in future studies. For this, we selected 28 lung and brain formalin-fixed paraffin-embedded (FFPE) samples from previous experiments performed by our research group. Histopathology revealed severe pulmonary broncho-interstitial pneumonia, mainly in animals inoculated via the IN route, accompanied by a strong acute inflammatory response (Iba1^+^ cells) and high levels of NiV RNA. Upregulation of proinflammatory cytokines (IL-6 and TNF) was also observed by ISH RNAscope technique in these animals. Neurological lesions, consisting of perivascular cuffing and meningitis, were observed mainly in animals inoculated via IP route. IHC results showed astrocytosis (GFAP^+^) and microgliosis (Iba1^+^) in the brain of these animals, together with mild levels of IL6 and TNF mRNA. These results have helped us to characterize the host-pathogen interaction in the golden Syrian hamster animal model of NiV infection that is being currently used in preclinical testing of antiviral and vaccine strategies. Techniques used in this study could be applied to the development and application of golden Syrian hamster models of other infections by henipaviruses, including Hendra virus (HeV), and other high consequence priority pathogens.

## 1 Introduction

Nipah virus (NiV), a single stranded negative sense RNA virus from the genus *Henipavirus*, can cause severe disease in a variety of species including humans (1–4). This pathogen is included in the WHO Research and Development Blueprint list of epidemic threats, due to the absence of efficient antivirals or vaccines for the use in humans (5).

Although NiV has only been reported to cause outbreaks in Asia during the last few years (6–8), this and other related viruses within the *Henipavirus* genus represent an important public health threat, as more than half of the global population are under the geographical range of its natural reservoir, the fruit bat (1, 9, 10). *Pteropus* spp. fruit bats are the reservoir for NiV; however, NiV infection has also been reported in a variety of animals species, such as pigs, horses, cows, cats and dogs, which may be involved in the transmission to humans as a zoonotic disease (1, 2, 4, 11). In pigs, the disease was originally called “porcine respiratory and encephalitis syndrome (PRES)” or “barking pigs syndrome” and observed morbidity can reach up 100% (12). The first outbreak of NiV infection in humans affected pig farmers from infected farms in Malaysia, with humans and pigs showing similar respiratory and neurological signs and lesions (13). Nowadays, the most common source of NiV infection in humans is the consumption of fresh date palm sap and its derivates contaminated with bat excretions (1, 4, 14, 15). A large outbreak occurred in Kerala (Southern India) in 2018, resulting in 17 fatalities and subsequent annual outbreaks have been taking place in the same region since then (6, 7, 16). NiV outbreaks also occur frequently in Bangladesh and Malaysia (4, 11, 14, 15, 17, 18). Phylogenetic studies have demonstrated the presence of two genetically differentiated viral strains, NiV-M (Malaysia) and NiV-B (Bangladesh), which vary in the virulence observed in the human cases during the recent outbreaks (1, 2).

Multiple NiV vaccine candidates, targeting NiV surface glycoprotein (G) and/or fusion (F) protein as immunogens, are currently at preclinical or early development stages (19–21). Recently, Pastor and collaborators (22) showed the efficacy of a dendritic cell-targeting NiV vaccine candidate in an African green monkey model after challenge with the NiV-B. However, to date there are no regulatory-approved treatments or vaccines to prevent devastating outbreaks. Moreover, other similarly pathogenic viruses from the same genus, such as the Hendra virus (HeV), also lack licensed vaccines and antivirals for use in humans; although there is a commercially available subunit vaccine for the use in horses approved in Australia (Equivac) (23).

NiV infection in fruit bats is asymptomatic (2); however, in humans it can cause a severe neurological and respiratory disease with a mortality rate of 75% (1–3, 9). Animal models that can mimic human NiV disease are critical for a better understanding of virus pathogenesis and for developing vaccine candidates. Several animal models have been developed, including mice, guinea pigs, ferrets, non-human primates and golden Syrian hamsters, among others, each with their own strengths and limitations (24, 25). The golden Syrian hamster is a well characterized model and has several advantages, such as the ease of procurement and handling together with the reduced costs associated with housing requirements (19–23, 26–29).

We have recently established the NiV golden Syrian hamster infection model at the UK Health Security Agency (UKHSA) which induces a highly similar disease to that observed in human cases, including respiratory and neurological signs and lesions (20).

Understanding the pathogenesis and disease dynamics in animal models is essential for developing therapies and vaccines. Although several studies have explored the mechanism of NiV infection, some aspects of the pathogenesis are still not well defined, such as how the virus enters the central nervous system (CNS). Thus, the aim of this study is to characterize the pathology and the host-virus interaction in the golden Syrian hamster model of NiV infection, focusing on the host inflammatory response which takes place in the main target organs (lung and brain) using multiple histopathological techniques: immunohistochemistry (IHC), *in-situ* hybridisation (ISH) and multiplex immunohistochemistry (mIHC).

## 2 Materials and methods

### 2.1 Animal experimentation and samples

Archived tissue samples from several animal experiments were used in this study (20, 30). These animal experiments were carried out at the United Kingdom Health Security Agency (UKHSA), Porton Down laboratory. All the experiments were carried out in accordance with the local legislation and institutional requirements and were compliant with the United Kingdom Scientific Procedures Act (Animals) 1986 and the United Kingdom Codes of Practice for the Housing and Care of Animal Used in Scientific Procedures, 1989; under the authority of a Project License PP3877532 granted by the UK Home Office. This project license to perform the animal experiments was approved following ethical review by the UKHSA’s Animal Welfare and Ethical Review Body (AWERB).

Sixty-three Golden Syrian hamsters of >6 weeks of age were purchased from Envigo (Hillcrest, UK). Hamsters were singly housed in cages in a half-suited rigid isolator within an ACDP Containment Level 4 (Biosafety Level 4) laboratory, with *ad libitum* access to food and water, and were divided into different groups. Animals were challenged with NiV (Malaysian strain; GenBank no. AF212302) provided by the Special Pathogens Branch of the Centers for Disease Control and Prevention, Atlanta, USA. Different routes of infection, challenge virus concentration and previous immunization status were applied, as shown in Table 1 [see (20, 30)]. In total twenty-eight animals were selected, including 4 animals from each group. Animals from group 5 were immunized using a prime boost regimen with NiV soluble glycoproteins (NiV sG) with 3 weeks between each vaccination and subsequent challenge (30). Animals from group 7 were inoculated with phosphate buffered saline (PBS), serving as a control group.

**Table 1:** Details of animal experiments and sample selection.

Group (*n* = 4)	Route of infection	Dose (TCID_50_)	Immunization	DPC	Source
Group 1	IN	10^5^	–	4 dpc (1/4) and humane endpoint (4–5 dpc) (3/4)	(20)
Group 2	IN	10^4^	–	Humane endpoint (4–6 dpc) (4/4)	(20)
Group 3	IP	10^3^	–	2 dpc (2/4) and humane endpoint (6 dpc) (2/4)	(20)
Group 4	IP	10^2^	–	21 dpc (1/4) and humane endpoint (6–7 dpc) (3/4)	(20)
Group 5	IP	10^2^	NiV sG	14 dpc (4/4)	(30)
Group 6	IP	10^2^	PBS	Humane endpoint (7–10 dpc) (4/4)	(30)
Group 7	IP	PBS	–	21 dpc (4/4)	(30)

A total of 28 golden Syrian hamsters were selected, including 4 animals in each group. Route of infection, dose, immunization and dpc. IN, Intranasal; IP, Intraperitoneal; dpc, days post challenge; PBS, phosphate buffered saline; NiV sG, Nipah virus and Hendra virus soluble glycoproteins.

After the infection, animals were checked twice a day for clinical signs (increased to 4 times a day after manifestation of clinical signs) and weighed/temperature checked daily. Animals were humanely euthanized under terminal anesthesia via inhaled halothane and with an overdose of pentobarbital administered intraperitoneally at the scheduled end of the study [2-, 4- and 21-days post-challenge (dpc) Table 1] or upon meeting pre-determined humane endpoint consisting of 20% weight loss or neurological signs. Samples from lung, liver, brain and spleen were collected during the necropsies for subsequent analysis.

### 2.2 Histopathological study

Tissue samples from lung, liver, brain and spleen were fixed by immersion in 10% neutral-buffered formalin (NBF) for 3 weeks and then routinely processed into paraffin wax. 4 μm sections were cut and stained with hematoxylin and eosin (H&E). Stained slides were digitalized using a Hamamatsu S360 digital slide scanner (Hamamatsu Photonics K.K., Shizuoka, Japan) and examined with the ndp.view2 software (Hamamatsu Photonics K.K., v2.8.24).

The severity of the lesions was recorded with a semi-quantitative score, previously described by our group (20, 30). Briefly, the severity of broncho-interstitial pneumonia in the lung, the presence of inflammatory cellular infiltrates in the liver and the presence of infiltrates and lymphoid depletion in the spleen and the presence of meningitis and perivascular cuffing in the brain was recorded as follows, 0 = within normal limits; 1 = minimal; 2 = mild; 3 = moderate and 4 = marked/severe. In the brain and spleen, the sum of both scored parameters for those organs was considered the cumulative score (20, 30).

### 2.3 NiV RNA and mRNAs cytokines *in-situ* hybridization

Samples were stained using the *in-situ* hybridization (ISH) RNAscope technique to identify NiV RNA. The staining was automatically performed in the Leica BOND-RXm (Leica Microsystems, Milton Keynes, United Kingdom). Briefly, slides were pre-treated with hydrogen peroxide for 10 min (min), target retrieval for 15 min (98–101°C), and protease plus for 30 min (40°C) (Advanced Cell Diagnostics, CA, USA). A NiV-specific probe (Cat No. 439258, Advanced Cell Diagnostics) was applied, and samples were incubated for 2 h at 40°C. Amplification of the signal was performed using the RNAscope 2.5 HD Detection Kit – RED (Advanced Cell Diagnostics) following the manufacturer instructions.

Additionally, the same ISH-RNAscope technique was applied manually to detect IL-6 mRNA (Cat No. 1062321-C1), TNF mRNA (Cat No. 1062341-C1) and IFNβ1 mRNA (Cat No. 1163061-C1) cytokines in lung and brain samples from selected groups (1, 4, 5, 6 and 7) considering the severity of the lesions. The technique was performed under the same conditions and following the manufacturer’s instructions [RNAscope 2.5. HD Detection Reagent – RED (Advanced Cell Diagnostics)]. Positive control tissues from previous experiments and adequate negative controls were also included in each ISH run.

After mounting using EcoMount (Biocare Medical, CA, USA), slides were digitally scanned with the Hamamatsu S360 digital slide scanner (Hamamatsu Photonics K.K). In order to quantify the presence of NiV RNA, slides were evaluated with the Nikon NIS-Ar software (Nikon, Instruments Inc., NY, USA) to calculate the percentage of positivity (percentage area positively stained). Due to the low level of the staining for cytokine mRNA (IL-6, TNF and IFNβ1), positive staining quantification was only performed in samples from the lung.

### 2.4 Immunohistochemistry (IHC) and multiplex IHC (mIHC)

#### 2.4.1 Immunohistochemistry (IHC)

Immunohistochemistry (IHC) was performed to characterize the cell components of lesions and inflammatory infiltrates using primary antibodies to mark T cells (CD3^+^) and pneumocyte type II/macrophages (Iba1^+^) within the lung and T cells (CD3^+^), microglia/macrophages (Iba1^+^) and astrocytes (GFAP^+^) within the brain. A summary of the IHC method is shown in Table 2. Briefly, immunostaining was performed on the Leica BOND-RXm (Leica Microsystems) using BOND Epitope Retrieval Solution 1 (ER1, pH 6.0) for 20 min at 95°C as antigen retrieval. After primary antibody incubation, immunostaining was carried out with the Bond polymer refine detection kit (Leica Microsystems). Finally, slides were routinely dehydrated and mounted using the Dako mounting medium (Agilent, Santa Clara, USA). To compare the results with the NiV ISH-RNAscope, IHC against the anti-NiV nucleoprotein antibody (Invitrogen, MA, USA) was performed following the same protocol and conditions (ER1, pH 6.0 for 20 min at 95°C) (Table 2). Negative reagent controls, consisting of replacement of primary antibody by normal serum (for polyclonal antibodies) or IgG isotype (for monoclonal antibody) at the same concentration that the target antibody, were added to each run to demonstrate the absence of false positive results (Supplementary Figure 1). Additionally, a supplementary negative control omitting primary antibody (“OMIT”) was also added to each run (Supplementary Figure 1). Positive control tissues from previous experiments carried out by our group were also included in each IHC run.

**Table 2:** Summary of immunohistochemical (IHC) methods: primary antibody details, dilution, blocking solution, antigen retrieval and source.

Primary antibody/Clone	Type of antibody	Dilution	Blocking solution	HIER	Source
CD3	Polyclonal	1:50	Superblock^1^	ER1 20 min^2^	Agilent, SA, USA
GFAP	Polyclonal	1:1000	Superblock^1^	ER1 20 min^2^	ThermoFisher Scientic, Massachusetts, USA
Iba1	Polyclonal	1:750	Superblock^1^	ER1 20 min^2^	FujiFilm Wako, Neuss, Germany
Anti-NiV nucleoprotein (clone, HL1436)	Monoclonal	1:200	Superblock^1^	ER1 20 min^2^	Invitrogen, MA, USA

HIER, heat-induced epitope retrieval. ^1^Superblock (TBS) Blocking Buffer (Thermo Scientific, USA); ^2^BOND Epitope Retrieval Solution 1 (pH 6.0).

IHC stained slides were scanned and subjected to digital image analysis to calculate the percentage of positively stained area using Nikon NIS-Ar software (Nikon). Additionally, in the lung the percentage area of CD3^+^ and Iba1^+^ within perivascular infiltrates was also evaluated by drawing the perivascular areas (as RIOs-regions of interests) in the whole lung using Nikon NIS-Ar software.

#### 2.4.2 Multiplex immunohistochemistry (mIHC)

A multiplex immunohistochemistry (mIHC) technique was developed to study the host-pathogen interaction of NiV in lung and brain. Selected samples from lung (group 6) and brain (group 5) with severe histopathological lesions and high viral RNA were sectioned at 4 μm. The same antibodies employed for IHC were used for mIHC (anti-NiV nucleoprotein, CD3, GFAP and Iba1 antibodies). Sections were stained using the Opal Polaris 7-Color automatication IHC kit (Akoya Biosciences, MA, USA, Cat No. NEL871001KT) for 5-plex staining. Staining was performed with the Leica BOND-RXm (Leica Microsystems). A summary of the mIHC technique and Opal-fluorochrome tag applied to each primary antibody are shown in Table 3. Slides were scanned using the multispectral camera of a PhenoImager® HT (Akoya Biosciences) and images were acquired using the PhenochartTM software (Akoya Biosciences).

**Table 3:** Summary of multiplex immunohistochemistry (mIHC) techniques: primary antibody and Opal tag detail.

Primary antibody	Primary antibody dilution	HIER	Opal	Opal dilution
Anti-NiV nucleoprotein	1:200	ER1 20 min^1^	Opal 520 (green)	1:150
CD3	1:50	ER1 20 min^1^	Opal 570 (yellow)	1:150
GFAP	1:200	ER1 20 min^1^	Opal 620 (orange)	1:150
Iba1	1:200	ER1 20 min^1^	Opal 690 (red)	1:150
–	–	ER2 20 min^2^	Dapi (blue)	1:10

HIER, heat-induced epitope retrieval. ^1^BOND Epitope Retrieval Solution 1 (pH 6.0). ^2^BOND Epitope Retrieval Solution 2 (pH 9.0).

All histopathology, ISH, IHC and mIHC studies were carried out in a ISO9001:2015 and GLP compliant laboratory.

## 3 Results

### 3.1 Histopathological lesions

**Figure 1:**
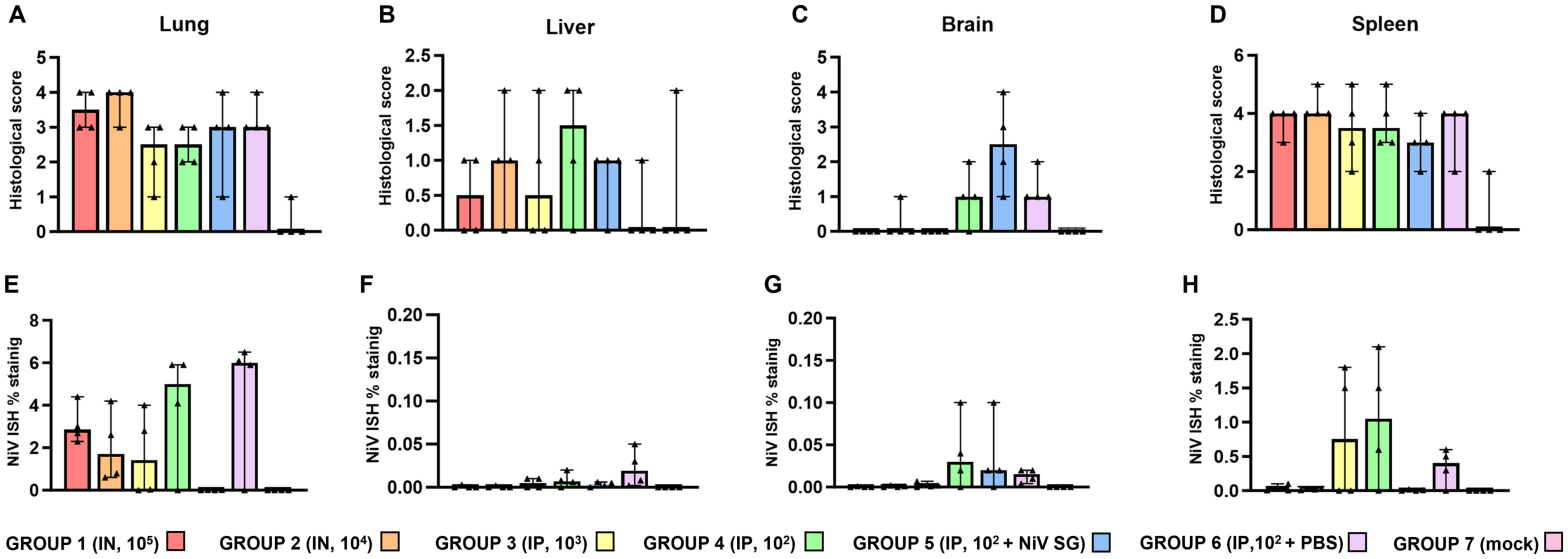
Quantitative readouts of histopathological score and *in-situ* hybridisation (ISH) RNAscope results from different experimental groups. Histopathological score in **(A)** lung, **(B)** liver, **(C)** brain, and **(D)** spleen. Digital image analysis (percentage area positively stained) of ISH in **(E)** lung, **(F)** liver, **(G)** brain, and **(H)** spleen. Data points show values from individual animals (black triangles) with columns and whisker plots denoting median with range. *N* = 4 animals per experimental group.

**Figure 2:**
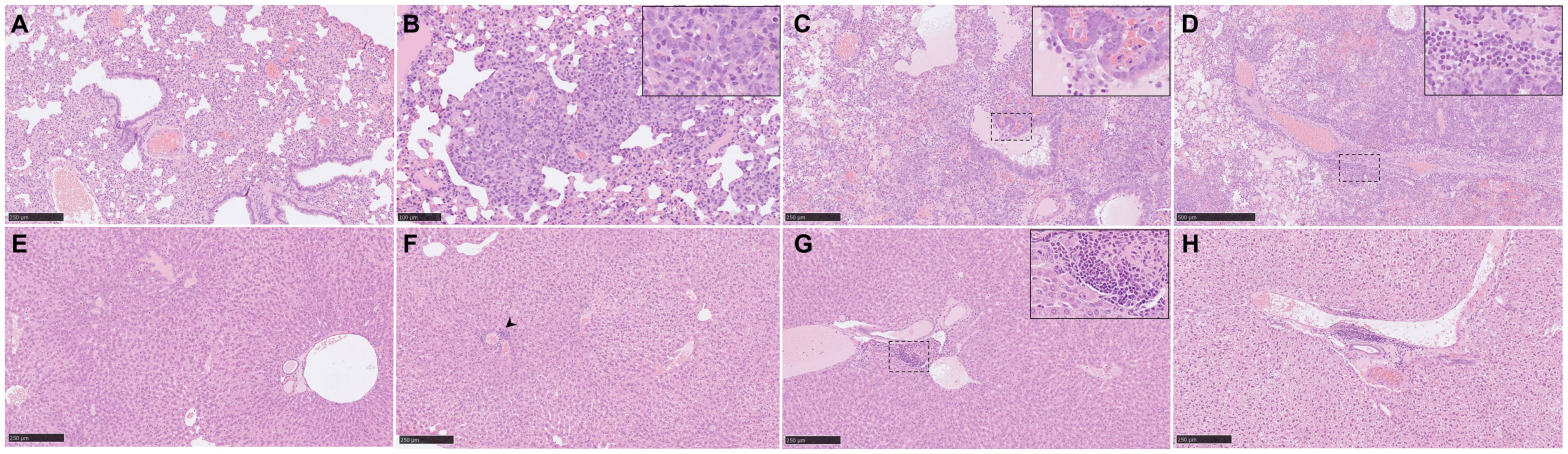
Representative histopathological lung and liver lesions from NiV-M pathology (H&E) in different experimental groups. **(A)** Lung section from an animal from group 3 showing moderate interstitial pneumonia characterized by thickening of the alveolar walls. **(B)** Lung section from an animal from group 5 showing moderate interstitial bronchopneumonia characterised by thickening of the alveolar walls and pneumocyte type II hyperplasia. Inset shows pneumocyte type II hyperplasia. **(C)** Lung section from an animal from group 1 showing severe broncho-interstitial pneumonia. Inset shows necrosis of alveolar and bronchiolar epithelium with heterophils, cell debris, alveolar macrophages, and oedema in the bronchiolar, and bronchial luminae. **(D)** Lung section from the same animal showing perivascular infiltration. Inset shows infiltration by mononuclear cells and heterophils. **(E)** Liver section from an animal from group 1 showing no histopathological lesions. **(F)** Liver section from an animal from group 5 showing the presence of an inflammatory infiltrate (arrowhead). **(G)** Liver section from an animal from group 3 showing the presence of an inflammatory infiltrate. Inset shows mononuclear cells and fewer heterophils. **(H)** Liver section from an animal from group 4 showing the presence of an inflammatory infiltrate. Scale bars a, c, e, f and g = 250 μm; b = 100 μm; and d = 500 μm.

**Figure 3:**
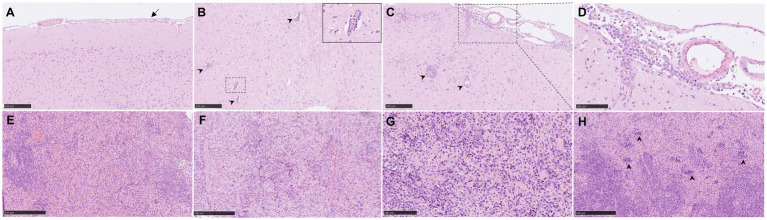
Representative histopathological brain and spleen lesions from NiV-M pathology (H&E) in different experimental groups. **(A)** Brain section from an animal from group 4 showing mild meningitis. **(B)** Brain section from an animal from group 5 showing perivascular cuffing (arrowheads). Inset shows higher magnification of a perivascular cuffing. **(C)** Brain section from an animal from group 5 showing severe meningitis and perivascular cuffing (arrowheads). **(D)** Higher magnification of the previous pictures showing mononuclear cells within the meningitis. **(E)** Spleen section from an animal from group 2 showing moderate degree of lymphoid depletion. **(F)** Spleen section from an animal from group 4 showing severe degree of lymphoid depletion. **(G)** Spleen section from an animal from group 6 showing tingible-body macrophages and apoptotic bodies. **(H)** Spleen section from an animal from group 6 showing severe degree of heterophil infiltration within the red pulp (arrowheads). Scale bars a, b, c, e and *f* = 250 μm; d and g = 100 μm.

Histopathological changes were only observed in organs from infected groups (Figures 1A–D, 2, 3). Histological examination from group 7 (negative control) is shown in Supplementary Figure 2.

#### 3.1.1 Histopathology of lung

The severity of the pulmonary lesions was similar in all infected groups (Figure 1A). Lesions consisted of moderate to severe multifocal to coalescing areas of broncho-interstitial pneumonia characterized by thickening of the alveolar walls (Figures 2A,B) and type II pneumocyte hyperplasia (Figure 2B, inset). In the most severe cases, necrosis of alveolar and bronchiolar epithelium was also observed together with heterophils, cell debris, alveolar macrophages, and mucus plugs filling the alveolar, bronchiolar, and bronchial luminae (Figure 2C, inset). Moreover, perivascular and peribronchial/bronchiolar infiltration by mononuclear cells and heterophils was also observed in the most severe cases (Figure 2D, inset).

#### 3.1.2 Histopathology of liver

No histopathological lesions were observed in most of the liver samples (Figure 2E). The main lesion observed was the presence of inflammatory cell infiltrates composed mainly by mononuclear and fewer polymorphonuclear cells (Figure 2F, arrowhead, Figures 2G,H). These cell infiltrates were frequently observed at the portal areas or surrounding the central veins of the hepatic lobules (Figures 2G,H).

#### 3.1.3 Histopathology of brain

In the brain samples the most frequently observed lesion was a mild meningitis composed mainly by mononuclear cells, observed in IP inoculated animals (Figure 1C and Figures 3A–D). The meningeal inflammatory cell infiltration was observed in all areas of the encephalon. Of note, in the brain from animals from group 5, a higher severity of the lesions was observed (Figure 1C), accompanied by perivascular cuffing of variable severity (composed mainly by mononuclear cells) principally located in the brainstem and mid-brain regions (Figures 3B,C, arrowheads and inset).

#### 3.1.4 Histopathology of spleen

Moderate to severe lymphoid depletion was observed in the spleen from all infected groups (Figures 1D, 3E,F). Tingible-body macrophages and apoptotic bodies (Figure 3G), together with heterophil infiltration mostly within the red pulp (Figure 3H, arrowheads) were present in the most severe cases.

### 3.2 NiV RNA distribution in tissues

**Figure 4:**
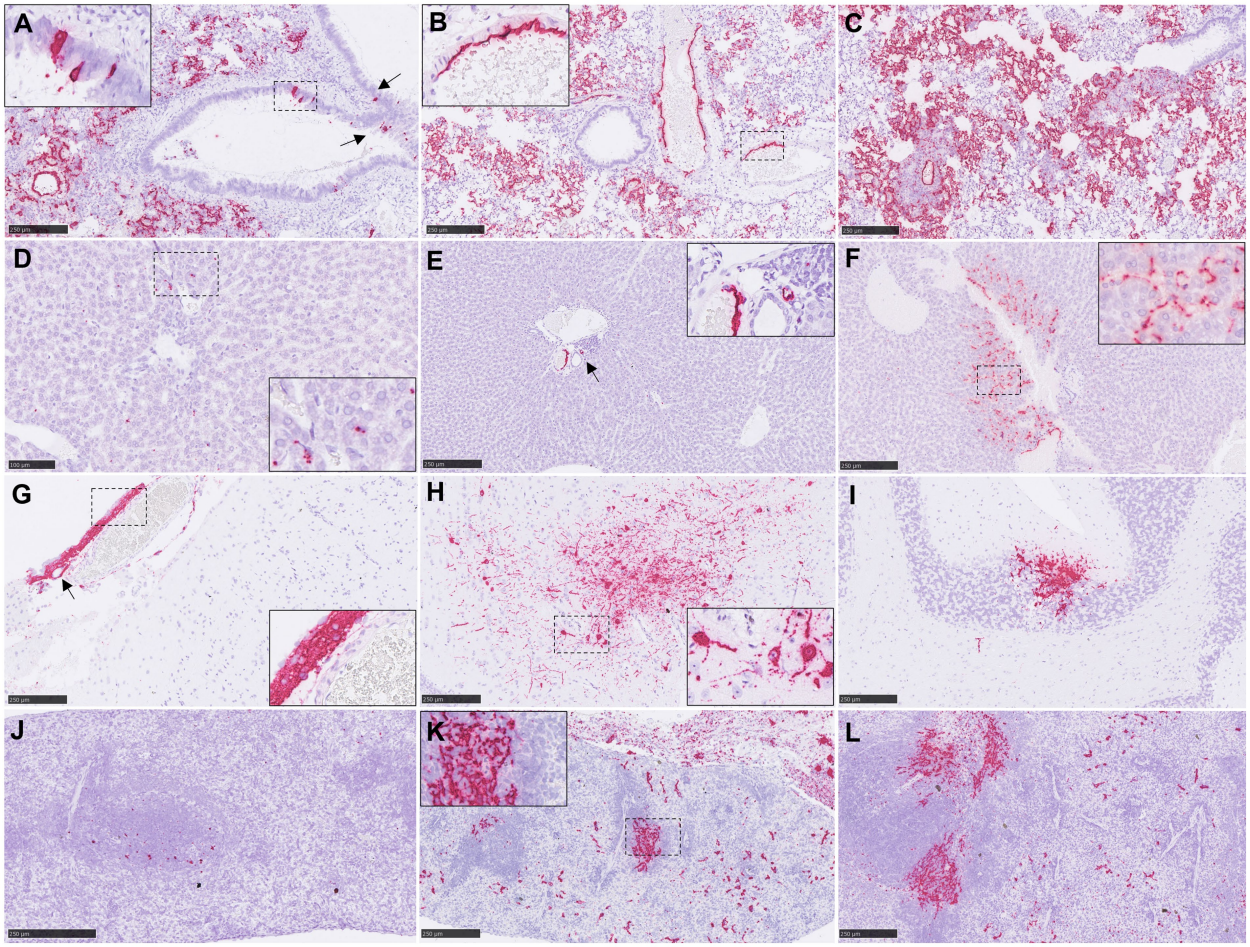
Representative pictures of *in-situ* hybridisation (ISH) RNAscope technique from NiV infected animals. **(A)** Lung section from an animal from group 1 showing viral RNA in areas of broncho-interstitial pneumonia and in epithelial cells from the bronchioli (arrows). Inset shows higher magnification of NiV^+^ epithelial cells. **(B)** Lung section from an animal from group 4 showing viral RNA in areas of broncho-interstitial pneumonia and in endothelial. Inset shows higher magnification of NiV^+^ endothelial cells. **(C)** Lung section from an animal from group 6 showing viral RNA in areas of broncho-interstitial pneumonia. **(D)** Liver section from an animal from group 5 showing viral RNA in Kupffer cells. Inset shows higher magnification. **(E)** Liver section from an animal from group 3 showing viral RNA in endothelial cells (arrow). Inset shows higher magnification. **(F)** Liver section from an animal from group 6 showing viral RNA in Kupffer cells, endothelial cells and liver sinusoids inset shows higher magnification. **(G)** Brain section from an animal from group 4 showing viral RNA in inflammatory cells and endothelial cells (arrow) from a meningitis area. Inset shows higher magnification. **(H)** Brain section from an animal from group 4 showing viral RNA neurons and neuropil within the mid-brain. Inset shows higher magnification of NiV^+^ neurons. **(I)** Brain section from an animal from group 4 showing NiV^+^ Purkinje cells and granule cells from the cerebellum. **(J)** Spleen section from an animal from group 2 showing scarce presence of viral RNA. **(K)** Spleen section from an animal from group 3 showing expression NiV RNA throughout the splenic parenchyma. Inset shows higher magnification. **(L)** Spleen section from an animal from group 4 showing expression of viral RNA throughout the splenic parenchyma. Scale bars a-c, e-l = 250 μm; d = 100 μm.

NiV RNA detected by ISH was not observed in organs from negative control animals (Figures 1E–H; Supplementary Figure 2). In the lung, tissues from group 6 showed the higher RNA expression in comparison with the rest of the groups (Figure 1E). A similar pattern of expression was observed between IN (groups 1 and 2) and IP inoculation (groups 3, 4, 5 and 6) within the inflammatory cell infiltrates in the areas of severe broncho-interstitial pneumonia and endothelial cells from blood vessels (Figures 4A–C). However, in IN inoculated animals, viral RNA was also detected within the bronchiolar and bronchial epithelial cells and airway exudates (Figure 4A, arrows and inset). Viral RNA in the liver was infrequently observed in some infected animals, especially from IP inoculated groups (Figures 1F, 4D–F). NiV RNA was located within Kupffer cells (Figure 4D, inset), endothelial cells from hepatic blood vessels (Figure 4E, inset) and liver sinusoids (Figure 4F, inset), occasionally associated to inflammatory infiltrates. In the brain, IP inoculated animals showed the higher percentage of positive area of NiV RNA (Figures 1G, 4G–I). Viral RNA was mainly detected in inflammatory cells (Figure 4G, inset) and endothelial cells (Figure 4G, arrow) within the areas of meningitis, and neurons and neuropil within the mid-brain regions and olfactory bulb (Figure 4H, inset). Additionally, viral RNA was observed in Purkinje cell neurons from the granular cell layers in the cerebellum (Figure 4I). A similar virus distribution among groups was observed in the spleen (Figures 1H, 4J–L). IP inoculated animals showed a diffuse expression of viral RNA throughout the splenic parenchyma, mainly in immune cells from the red pulp (Figure 4K, inset and Figure 4L).

### 3.3 Cell populations within pulmonary lesions

**Figure 5:**
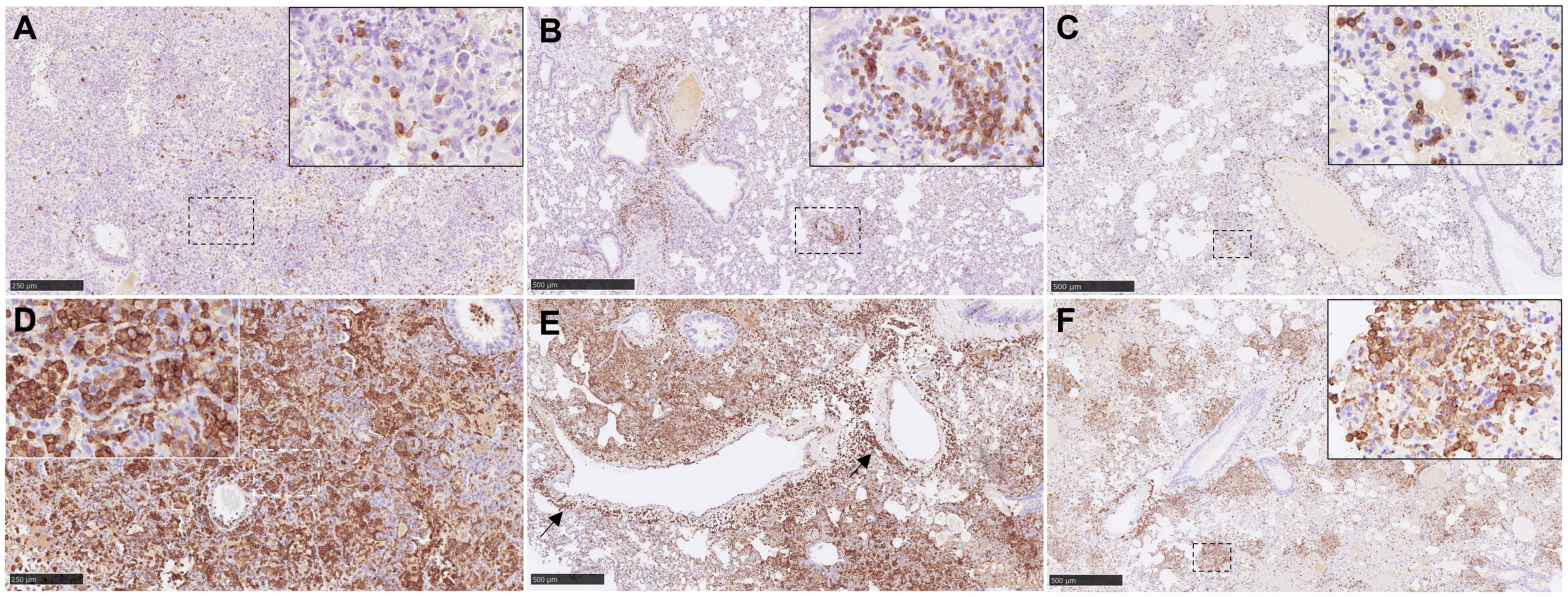
Representative pictures of immunohistochemistry (IHC) against CD3 and Iba1 in lung from NiV infected animals. **(A)** CD3 IHC from an animal from group 2 showing scattered T lymphocytes in areas of severe broncho-interstitial pneumonia. Inset shows CD3 IHC in the cell membrane of T lymphocytes. **(B)** CD3 IHC from an animal from group 5 showing CD3^+^ cells infiltrate at perivascular level. Inset shows higher magnification. **(C)** CD3 IHC from an animal from group 4 showing scattered T lymphocytes in areas of moderate broncho-interstitial pneumonia. Inset shows higher magnification of T lymphocytes. **(D)** Iba1 IHC from an animal from group 1 showing intense Iba1 staining in areas of severe broncho-interstitial pneumonia. Inset shows Iba1 staining in the cell membrane of alveolar macrophages. **(E)** Iba1 IHC from an animal from group 1 showing macrophage infiltrates at perivascular level. **(F)** Iba1 IHC from an animal from group 4 showing Iba1 staining in areas of multifocal broncho-interstitial pneumonia. Inset shows higher magnification of Iba1^+^ macrophages. Scale bars a and d = 250 μm; b, c, e and *f* = 500 μm.

CD3^+^ staining detected T cells diffusely scattered within the areas of broncho-interstitial pneumonia (Figures 5A,C). No major differences were observed between the groups, with low levels of CD3^+^ cells in all NiV-inoculated groups. Interestingly, lung from some animals showed perivascular infiltrates with a major CD3^+^ T cell component (Figures 5B, inset and 6B).

**Figure 6:**
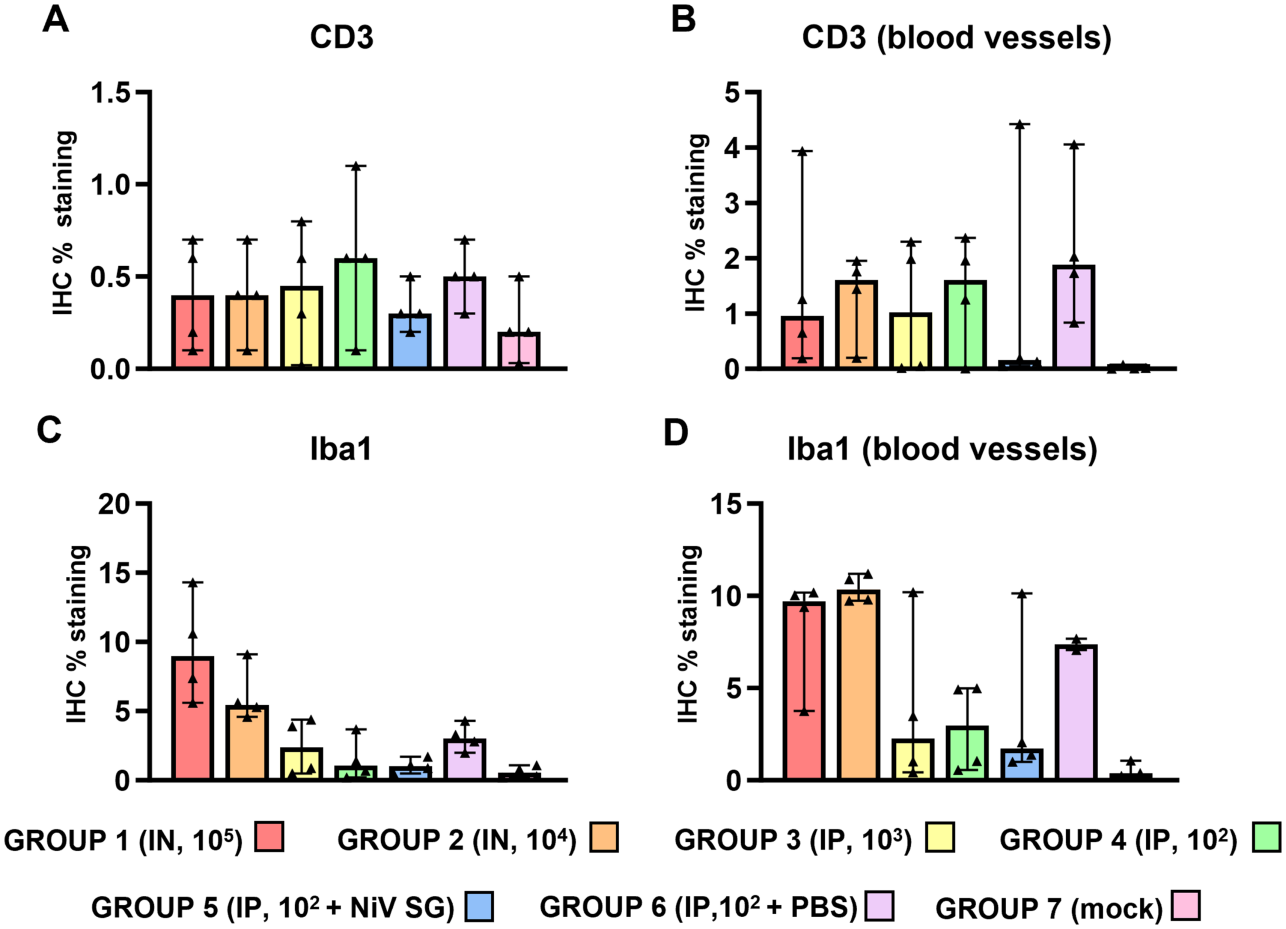
Quantitative results of CD3 and Iba1 immunohistochemistry (IHC) in lung parenchyma and at perivascular level from different experimental groups. **(A)** Graph represents quantification of CD3 IHC in lung. **(B)** Graph represents quantification of CD3 IHC at perivascular level. **(C)** Graph represents quantification of Iba1 IHC in lung. **(D)** Graph represents quantification of Iba1 IHC at perivascular level. Data points show values from individual animals (black triangles) with columns and whisker plots denoting median with range. *N* = 4 animals per experimental group.

Iba1^+^ cells (macrophages/type II pneumocytes) were the most predominant cells within the areas of broncho-interstitial pneumonia (Figure 6C), with higher frequency in the lungs from IN inoculated animals (Figures 5D,E). Iba1^+^ staining was detected on the cell membrane of alveolar macrophages (Figures 5D,F, insets). Interestingly, a perivascular pattern of expression was observed, especially in the IN inoculated animals (groups 1 and 2) (Figure 5E, arrows and Figure 6D). A small percentage of CD3^+^ and Iba1^+^ cells were observed in the lungs from negative control animals (Supplementary Figures 3A,B).

**Figure 7:**
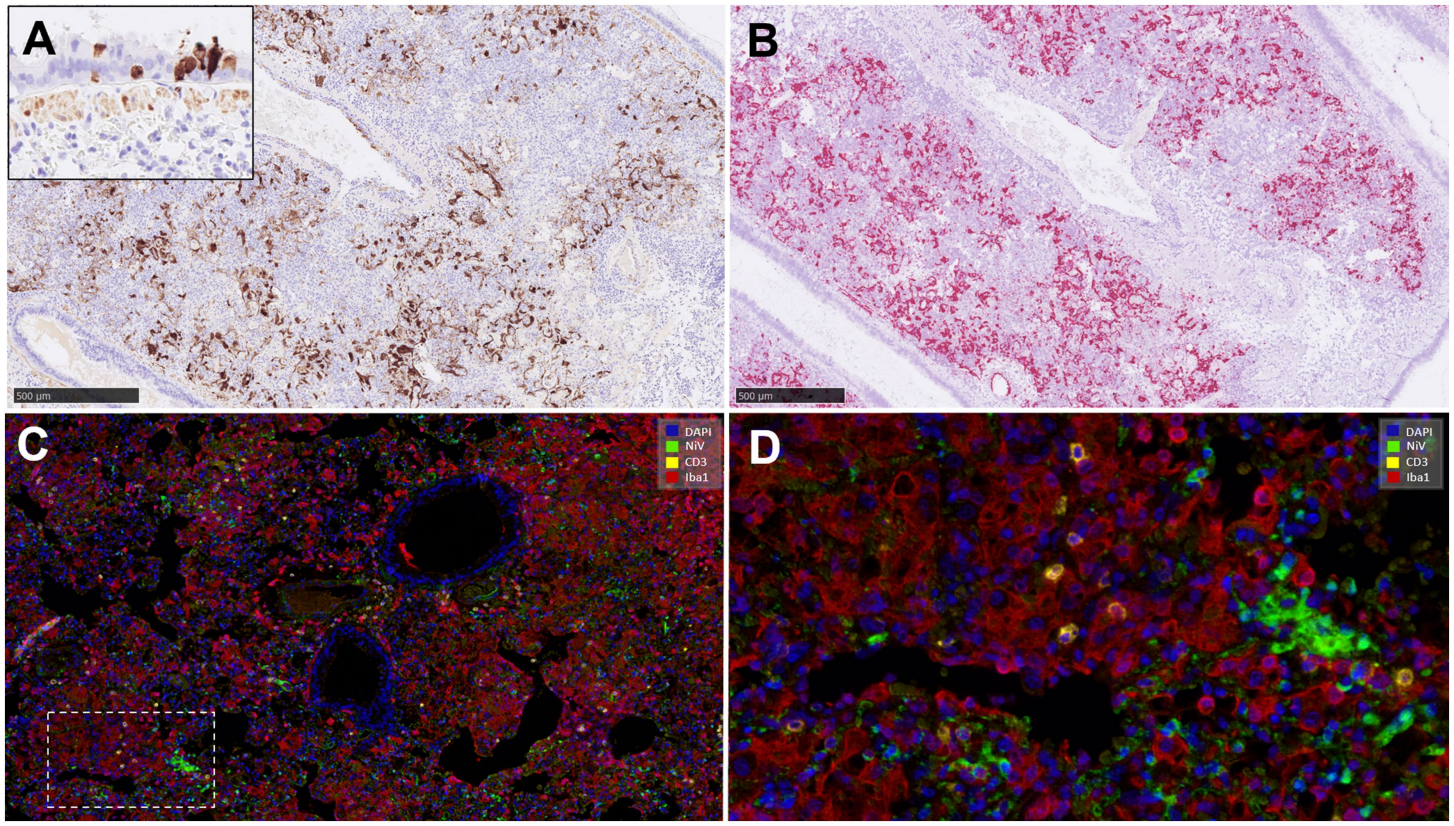
Representative pictures of immunohistochemistry (IHC) against NiV nucleoprotein compared with the NiV *in-situ* hybridisation (ISH) RNAscope technique, and multiplex immunohistochemistry (mIHC) in lung. **(A)** Representative picture from an animal from group 2 showing NiV^+^ staining in areas of interstitial broncho-interstitial pneumonia. Inset shows NiV^+^ epithelial cells from an animal from group 1. **(B)** Same field as **(A)** performed using ISH-RNAscope analysis showing similar positivity with both techniques. **(C)** MIHC on an animal from group 4 showing 4plex staining, blue colour DAPI, green colour NiV^+^ (opal 520), yellow colour CD3^+^ (opal 570) and red colour Iba1^+^ (opal 690). **(D)** Higher magnification of the previous picture. Scale bars a and b = 500 μm.

The distribution of NiV nucleoprotein positive stain was similar to that obtained for NiV RNA staining by ISH-RNAscope analysis (Figures 7A,B) with the same pattern of expression between the different groups (Supplementary Figure 4). However, despite the efforts in the development of the protocol, some non-specific staining could be observed in the smooth muscle cells from the blood vessels, which need to be taken into consideration when using this antibody. No specific staining was detected in samples from negative control animals (group 7) (Supplementary Figure 3C). In samples from IN inoculated animals (groups 1 and 2), the presence of NiV IHC^+^ cells was observed (Figure 7A, inset), similar to that observed with the ISH-RNAscope analysis (Figure 4A, inset).

### 3.4 Multiplex immunohistochemistry (mIHC) within pulmonary lesions

MIHC showed only a small amount of CD3^+^ cells in comparison with the amount of IBA1^+^ cells in the same area (Figures 7C,D). Infiltrates of Iba1^+^ cells within the areas of broncho-interstitial pneumonia were co-located with NiV staining, showing a high level of NiV infection in macrophages (Figure 7D).

### 3.5 Proinflammatory cytokines expression in the lung

**Figure 8:**
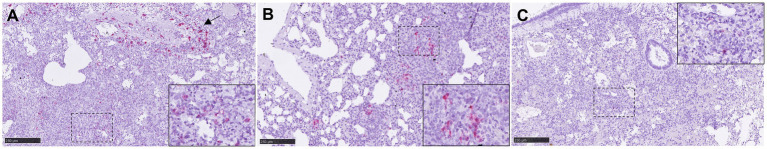
Representative pictures of *in-situ* hybridisation (ISH) RNAscope technique of IL-6, TNF and IFNβ1 mRNA in lung. **(A)** Representative picture from an animal from group 1 showing high numbers of IL-6 mRNA cells in areas of severe interstitial broncho-interstitial pneumonia and at perivascular level (arrow). Inset shows higher magnification. **(B)** Representative picture from an animal from group 5 showing TNF mRNA staining in areas of interstitial pneumonia. Inset shows higher magnification. **(C)** Representative picture from an animal from group 1 showing low expression of IFNβ1 mRNA. Inset shows higher magnification. Scale bars = 250 μm.

IL-6 was the most cytokine expressed at transcript level, mainly in IN inoculated animals (group 1) (Supplementary Figure 4). IL-6 mRNA was associated with inflammatory cells within areas of broncho-interstitial pneumonia (Figure 8A, inset) and around the blood vessels. Smaller quantities of IL-6 mRNA were observed in the lung from the rest of groups, but higher in comparison with the negative controls (group 7) (Supplementary Figures 3D–F, 5A). Small amounts of TNF mRNA were detected in the lung from all infected animals (Supplementary Figure 5B), associated to macrophage-like and type II pneumocyte-like cells. Positive TNF mRNA cells were associated with areas of broncho-interstitial pneumonia (Figure 8B). The presence of IFNβ1 mRNA in lungs from infected animals was scarce (Supplementary Figure 5C), with positive inflammatory cells only occasionally observed (Figure 8C, inset).

### 3.6 Cell populations within the brain

**Figure 9:**
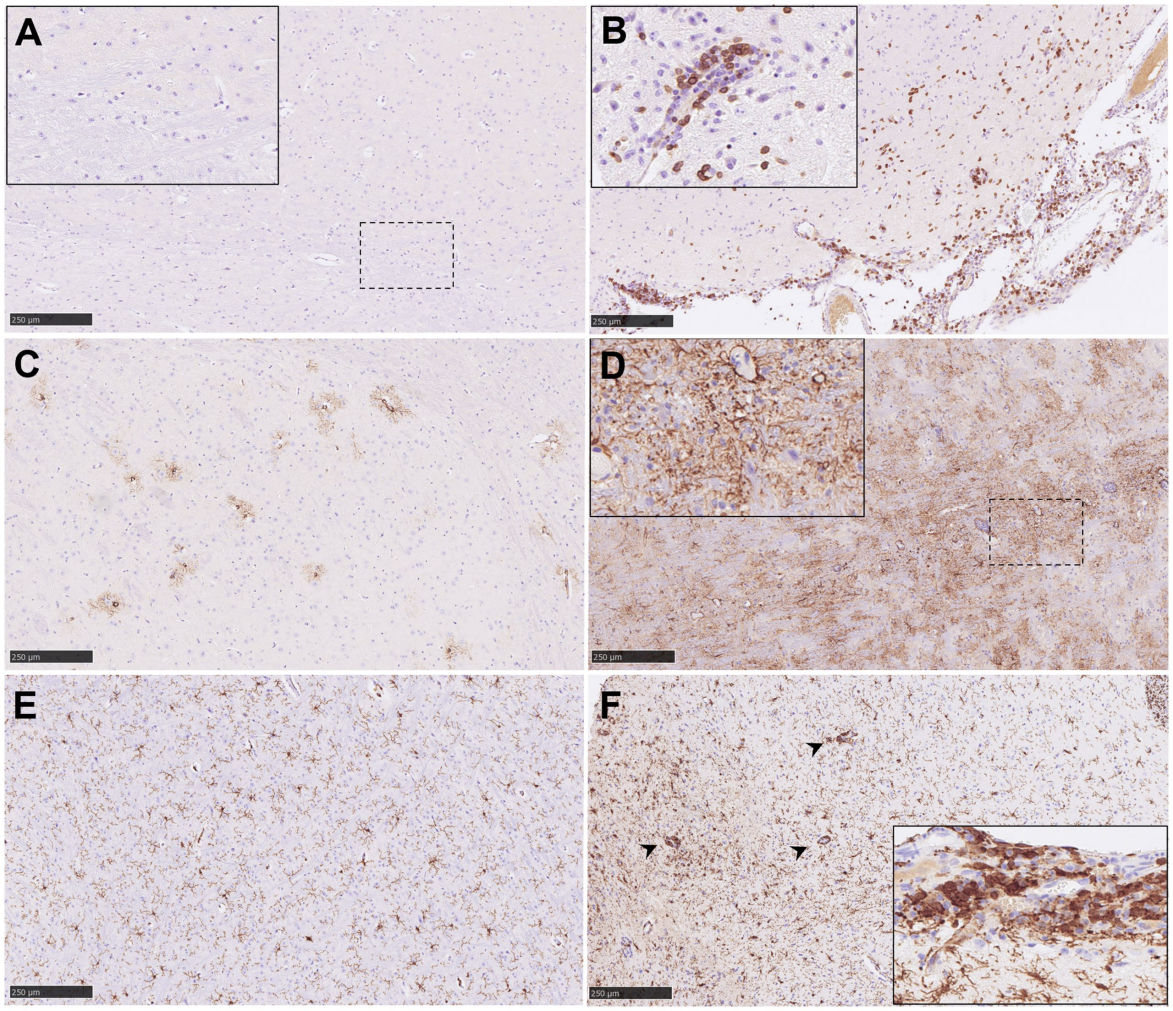
Representative pictures of immunohistochemistry (IHC) against CD3, GFAP and Iba1 in brain from NiV infected animals. **(A)** CD3 IHC from an animal from group 2 showing no CD3^+^ T lymphocytes in the mid-brain. Inset shows higher magnification. **(B)** CD3 IHC from an animal from group 5 showing a high number of CD3^+^ T lymphocytes in the mid-brain and meninges. Inset shows perivascular cuffing with CD3^+^ T lymphocytes in the mid-brain from an animal from the same group. **(C)** GFAP IHC from an animal from group 1 showing few GFAP^+^ astrocytes in the mid-brain. **(D)** GFAP IHC from an animal from group 1 showing high number of GFAP^+^ astrocytes in the mid-brain. **(E)** Iba1 IHC from an animal from group 1 showing few Iba1^+^ macrophages in the mid-brain. **(F)** Iba1 IHC from an animal from group 5 showing a high number of Iba1^+^ macrophages in the mid-brain. Arrow heads shows perivascular cuffing with Iba1^+^ macrophages. Inset shows meningitis with a high number of Iba1^+^ macrophages. Scale bars = 250 μm.

**Figure 10:**
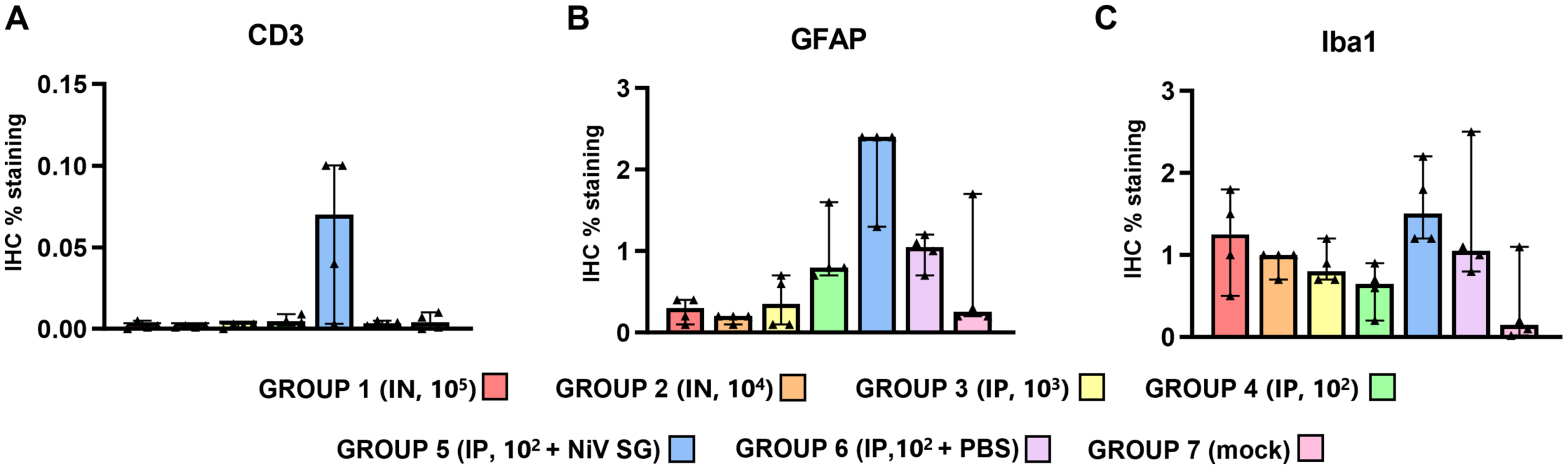
Quantitative results of CD3, GFAP and Iba1 immunohistochemistry (IHC) in brain. **(A)** Graph represents quantification of CD3 IHC in brain. **(B)** Graph represents quantification of GFAP IHC in brain. **(C)** Graph represents quantification of Iba1 IHC in brain. Data points show values from individual animals (black triangles) with columns and whisker plots denoting median with range. *N* = 4 animals per experimental group.

Results from the IHC quantification of CD3^+^, GFAP^+^ and Iba1^+^ cells are represented in Figure 8. CD3^+^ cells were mostly detected within perivascular cuffs (Figure 9B, inset) and meninges (Figure 9B) of the brain from IP inoculated animals from group 5 (Figures 9A,B, 10A). Moreover, scattered CD3^+^ cells were detected distributed diffusely within the mid-brain from these animals. No CD3^+^ staining was observed in PBS-inoculated control animals (Supplementary Figure 3G). IP inoculated animals (groups 4, 5 and 6) showed higher GFAP^+^ staining (Figure 10B) associated with astrocytosis. (Figures 9C,D), being more evident in the areas of meningitis and encephalitis and associated with perivascular cuffing (Figure 9D, inset). Higher expression of Iba1^+^ staining was found in the brain from all infected animals in comparison with the PBS control group (Supplementary Figure 3I; Figures 9E,F, 10C). Iba1^+^ staining was detected in the cytoplasm of macrophages/microglia, diffusely distributed throughout the brain from infected animals (Figures 9E,F), and also associated with inflammatory cell infiltration within the meninges (Figure 9F, inset) and perivascular cuffing (Figure 9F, arrowheads) in the most severe cases.

### 3.7 Multiplex immunohistochemistry (mIHC) within meningitis and encephalitis

**Figure 11:**
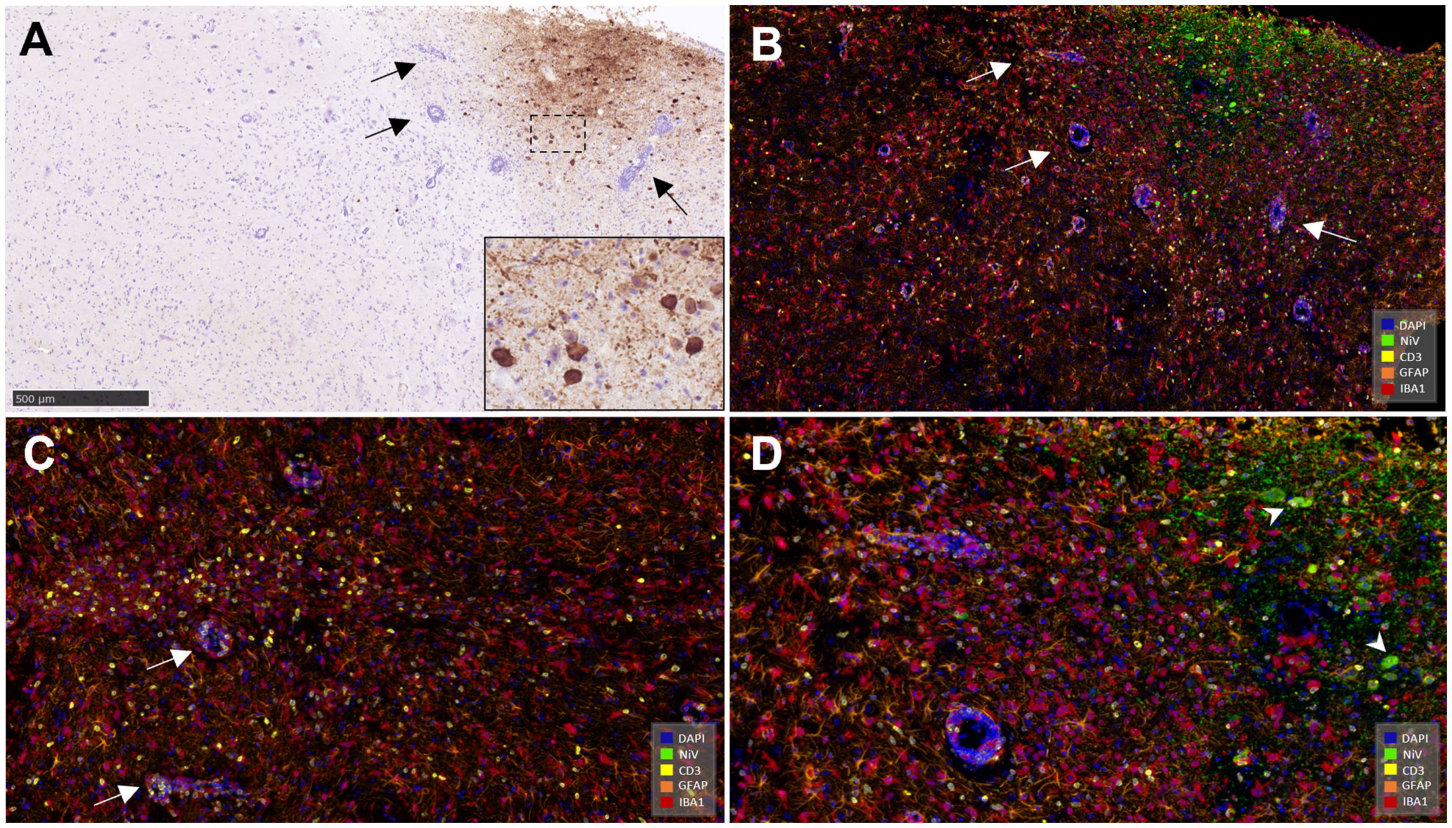
Representative pictures of immunohistochemistry (IHC) against NiV nucleoprotein and multiplex immunohistochemistry (mIHC) in brain. **(A)** Representative picture from an animal from group 5 showing NiV^+^ staining in the mid-brain. Arrows show perivascular cuffing. Inset shows NiV^+^ neurons. **(B)** Same field as **(A)** performed using mIHC showing similar virus positivity (opal 520, green colour) and the simultaneous expression of the rest of the markers in the mid-brain. NiV infected areas were infiltrated by a high number of astrocytes (GFAP^+^, opal 620, orange colour) and microglia (Iba1^+^, opal 690, red colour) and fewer T lymphocytes (CD3^+^, opal 570, yellow colour). Blue colour denotes DAPI staining. Arrows show perivascular cuffing. **(C)** Another field from the same sample showing a large quantity of CD3^+^ cells (yellow colour) and perivascular cuffing composed mainly by Iba1^+^ (red colour) cells and CD3^+^ cells. **(D)** Higher magnification of picture **(B)** showing the 5plex staining. Arrowheads shows NiV^+^ infected neurons. Scale bar a = 500 μm.

Similar NiV IHC and ISH-RNAscope staining in the brain allowed the validation of the anti-NiV primary antibody for its use in mIHC (Figures 11A,B). The areas with heavy presence of NiV showed severe astrocytosis (GFAP^+^ cells) and microgliosis (Iba1^+^) with scattered T cells (CD3^+^) (Figures 11B,D). Perivascular cuffing was observed within and surrounding the areas in the presence of NiV and was composed mainly of microglia/macrophages (Iba1^+^) and CD3^+^ T cells (Figures 11B,C). Large quantities of CD3^+^ cells were observed in some perivascular cuffs. NiV staining was detected in neuronal soma (Figure 11D, arrowheads), and the neuropil associated to neuronal extensions and glial cells.

### 3.8 Proinflammatory cytokine expression in the brain

**Figure 12:**
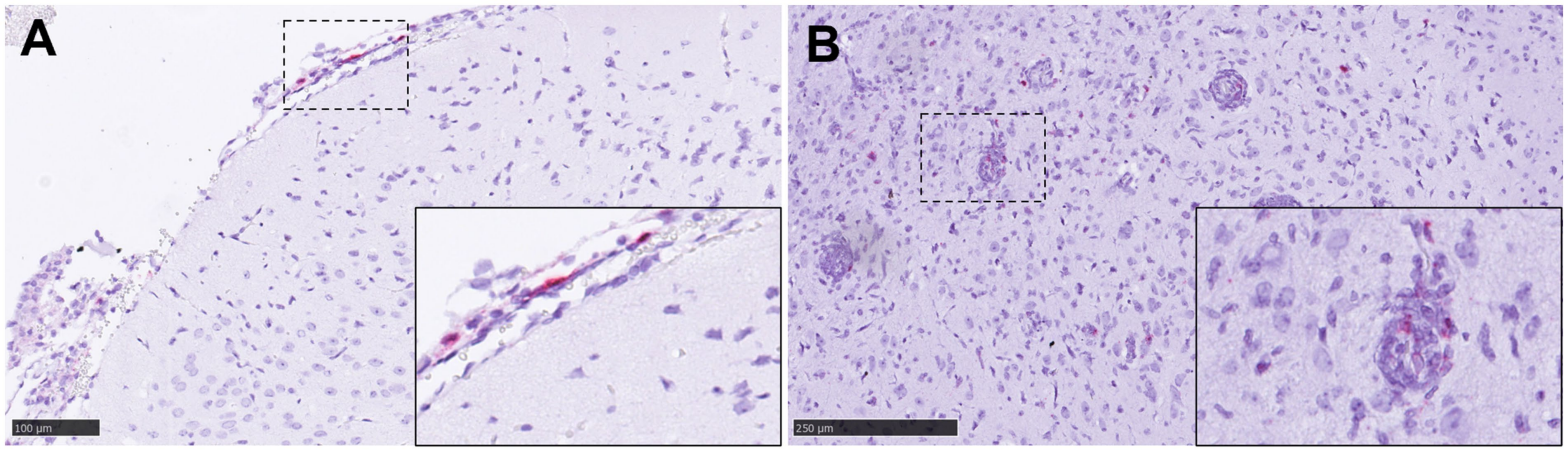
Representative pictures of *in-situ* hybridisation (ISH) RNAscope technique of IL-6 and TNF mRNA in brain. **(A)** Representative picture from an animal from group 6 showing IL-6 mRNA staining in an area of meningitis. Inset shows higher magnification. **(B)** Representative picture from an animal from group 5 showing TNF mRNA staining in areas encephalitis and perivascular cuffing. Inset shows higher magnification of a perivascular cuffing with some TNF^+^ mRNA cells. Scale bars a = 100 μm and b = 250 μm.

Due to the low level of staining, quantification of cytokine mRNA in the brain was not performed. Only few IL-6 mRNA^+^ inflammatory cells were found in the areas of meningitis from some IP inoculated animals (Figure 12A). Additionally, TNF mRNA^+^ cells were detected diffusely distributed within the brain and the perivascular inflammatory infiltration (Figure 12B, inset). IFNβ1 mRNA was not detected.

## 4 Discussion

NiV is included within the priority list of pathogens with pandemic potential with increasing attention on the development of medical countermeasures (31, 32). NiV may induce a severe respiratory disease with high mortality rates, ranging from 40 to 75%, and neurological sequelae in some individuals that can last for long periods of time (1, 2, 4, 15). The wide distribution of the principal NiV animal reservoir (*Pteropus* spp. fruit bats), together with the potential for direct transmission to humans or through a large range of domestic animals, and the lack of licensed vaccines makes NiV a major threat to public health (1, 2, 9). Moreover, direct person-to-person transmission has also been documented (1, 17, 33).

In this study, a golden Syrian hamster animal model was used to investigate the pathogenesis of NiV infection, as this animal species can display both respiratory and neurological disease, similar to that observed in humans (19–29). Most humans suffering from NiV infection show primarily respiratory symptoms (6, 7, 13, 16–18); for this reason, the IN inoculation route in animals might appear to more closely resemble the natural route of infection in humans. However, previous studies have shown that IP inoculation results in more consistent disease progression, including neurological signs (19, 20, 22, 28). Although other studies have described the histopathology and viral distribution in the golden Syrian hamster model (19–23, 27–29), a thorough description of the histopathology, cellular populations involved in the inflammatory response in target organs (lung and brain), and the host-pathogen interaction is lacking. In this study, we describe the microscopic lesions observed in the golden Syrian hamster model after IN and IP inoculation with different doses of NiV-M, the cell composition of the pulmonary and the CNS lesions and the expression of proinflammatory cytokines *in-situ* using a combination of classical histopathological techniques, IHC, ISH and multiplex IHC.

We have used archived material from previous studies carried out in our laboratory (20, 30) to maximize the use of animals in experimental research following the 3Rs recommendations. We have developed animal models for other high consequence pathogens such as SARS-CoV-2 or Influenza A virus using the golden Syrian hamster, developing IHC and ISH techniques for this animal species that can be applied in a variety of studies (34–36).

Although some histopathological lesions were observed in the liver and spleen, the main lesions were present in the lung and the CNS brain, confirming that both are the main target organs for NiV infection (13, 19, 22, 23, 27–29). The pulmonary lesions consisted of multifocal moderate to severe broncho-interstitial pneumonia, characterized by thickening of the alveolar wall mainly due to macrophage infiltration and type II pneumocyte hyperplasia (Iba1^+^) and small numbers of T cells (CD3^+^) and heterophils. These lesions were quite severe in IN inoculated animals, which also showed the presence of large quantities of NiV RNA in the areas of lesions associated with respiratory epithelial cells and inflammatory infiltrates, as observed in a variety of acute viral infections in the lung (19, 29, 35, 37–39). NiV has been documented to infect bronchiolar epithelial cells, type I pneumocytes and alveolar macrophages as early as 8 h after inoculation (19). In our study, we also observed a significant upregulation of proinflammatory cytokines in the lung, through analysis of the expression of cytokine mRNA by ISH. This “cytokine storm” has also been reported in other acute viral infections in animal models (40). IL-6 can be induced by the infection of the epithelium (3, 41) and upregulation of this cytokine has been observed by qRT-PCR in NiV infection (22, 29).

Endothelial cells displayed high rates of infection in both IN and IP infected animals, showing a high tropism of NiV for this cell type (21, 22, 28). Baseler et al. (28) demonstrated that the endotheliotropism of NiV is for arterioles and arteries rather than veins in the lung, which correlates with the expression of NiV receptor Ephrin B2. Although Wong and collaborators (21) reported no significant differences regarding the severity and the spectrum of pathological lesions between IN and IP inoculated animals, we have observed lower severity of lung lesions in IP compared to IN inoculated animals, with a less severe acute inflammatory response (a smaller number of Iba1^+^ macrophages), although high percentages of NiV infected cells were detected by ISH. This finding, together with the fact that histopathological lesions accompanied by high NiV RNA were found in the brain of IP inoculated animals suggest a faster organic NiV distribution through IP compared with IN inoculation (20). In IP inoculated animals, NiV can reach the systemic circulation directly without the need to penetrate the aerodigestive epithelial barrier (21), which would explain the presence of larger quantities of NiV detected in the rest of the organs. IN inoculated animals exhibit severe pulmonary clinical signs and lesions that meet the humane endpoint before the virus can sustain significant infection in other organs like the brain. This is contrary to the study performed by Wong and coauthors, where animals inoculated through the IN route showed longer survival rates (21). These discrepancies could be associated to the intrinsic differences between each experimental setting, including inoculation dose, inoculum volume or others.

NiV gets access to the blood stream through the respiratory epithelium and disseminates to endothelial cells in later stages of the disease (3, 41, 42). However, how NiV is disseminated to vasculature system and rest of the tissues, such as the CNS, is still poorly understood and viraemia is not detectable in many NiV models, including the ones we have used in this study (20, 30). A possible explanation is that NiV spreads via leukocytes, without being productively infected, and from leukocytes to endothelial cells for dissemination in different organs as proposed by Mathieu et al. (2011) (42). In IN infected hamsters, Munster and collaborators (2012) (27) showed the progression of NiV from the olfactory epithelium to the CNS, coinciding with the appearance of respiratory disease, suggesting a simultaneous entry of NiV into the CNS and lung.

Although Wong and collaborators (21) did not observe brain histopathological differences between IN and IP inoculation, we have only observed neuropathology (meningoencephalitis) in IP inoculated animals. Interestingly, those animals inoculated with the lower dose IP (10^2^ TCID_50_) were the ones that developed neurological lesions, in agreement with what was observed by Rockx and coauthors (2011) (29). These authors also demonstrated the disruption of the blood–brain barrier together with the upregulation of some proinflammatory cytokines such as TNF and IL-β1, which could be due to NiV replication in the brain (29). We have included animals from a failed vaccine candidate group (group 5), which presented striking neurological lesions (30). These lesions consisted of a meningoencephalitis with moderate to severe perivascular cuffing associated with the presence of NiV RNA, IL-6 and TNF mRNA. The perivascular cuffing (composed of CD3^+^ and Iba1^+^ cells) observed within these animals, points to the endothelial route as the mechanism of dissemination (42, 43). IHC results have shown that astrocytosis and microgliosis are present in infected animals, and these cells could be the main upregulators of proinflammatory cytokines in the brain since they are the main immune effectors cells in the CNS (43).

We have used ISH RNAscope analysis to study viral distribution in the different organs due to the high sensitivity and specificity of this technique (44, 45). Moreover, we have developed an IHC technique to detect NiV nucleoprotein in tissue section that also allows us to study the interaction of the virus with cell populations in the lung and brain using mIHC.

In conclusion, we have developed and applied an array of histopathological techniques to characterize the lesions and the local immune response in the lung and brain of NiV golden Syrian hamster animal model. These techniques will enable us to further characterize the immunological and protective responses against NiV infection after immunization with candidate vaccines. Moreover, these techniques can be applied to the development and application of golden Syrian hamster models for other henipavirus infections, such as Hendra virus, and other high consequence disease models.

## Data Availability

The raw data supporting the conclusions of this article will be made available by the authors, without undue reservation.
