# Supplementary material for: Pathology and host-pathogen interactions in a golden Syrian hamster model of Nipah virus infection
Source: Front Vet Sci. 2025 Mar 7;12:1518358. doi: 10.3389/fvets.2025.1518358 (PMC11926554; doi:10.3389/fvets.2025.1518358)
Supplement: Supplementary file 1 [file Supplementary_file_1.docx]

Supplementary Material

# Supplementary figures


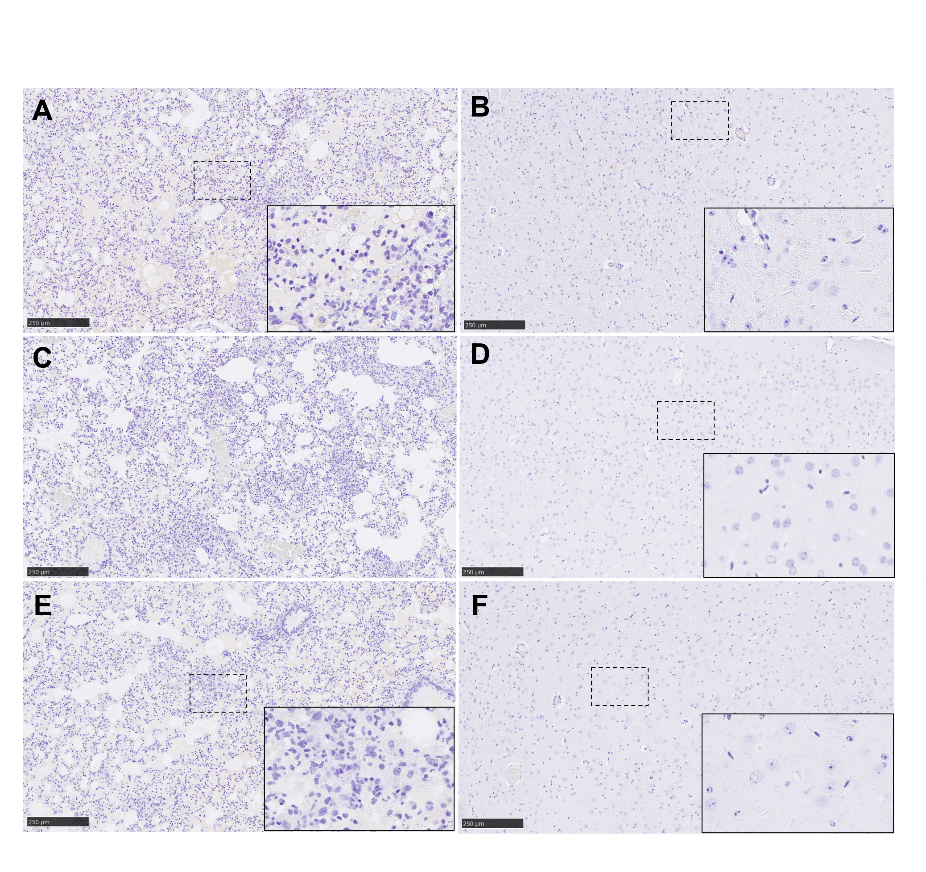


**Supplementary figure 1. Representative images from immunohistochemistry negative reagent controls. (a).** Lung section showing no specific staining. The primary antibody has been replaced by normal serum (control for polyclonal antibody target runs). Inset shows higher magnification. **(b)** Brain section showing no specific staining. The primary antibody has been replaced by normal serum (control for polyclonal antibody target runs). Inset shows higher magnification. **(c)** Lung section showing no specific staining. The primary antibody has been replaced by IgG isotype control (control for monoclonal antibody target runs). **(d)** Brain section showing no specific staining. The primary antibody has been replaced by isotype control (control for monoclonal antibody target runs). Inset shows higher magnification. **(e)** Lung section showing no specific staining. The primary antibody has been replaced by wash buffer (phosphate buffered saline; “OMIT”). Inset shows higher magnification. **(f)** Brain section showing no specific staining. The primary antibody has been replaced by wash buffer (phosphate buffered saline; “OMIT”). Inset shows higher magnification. Scale bars = 250 µm.


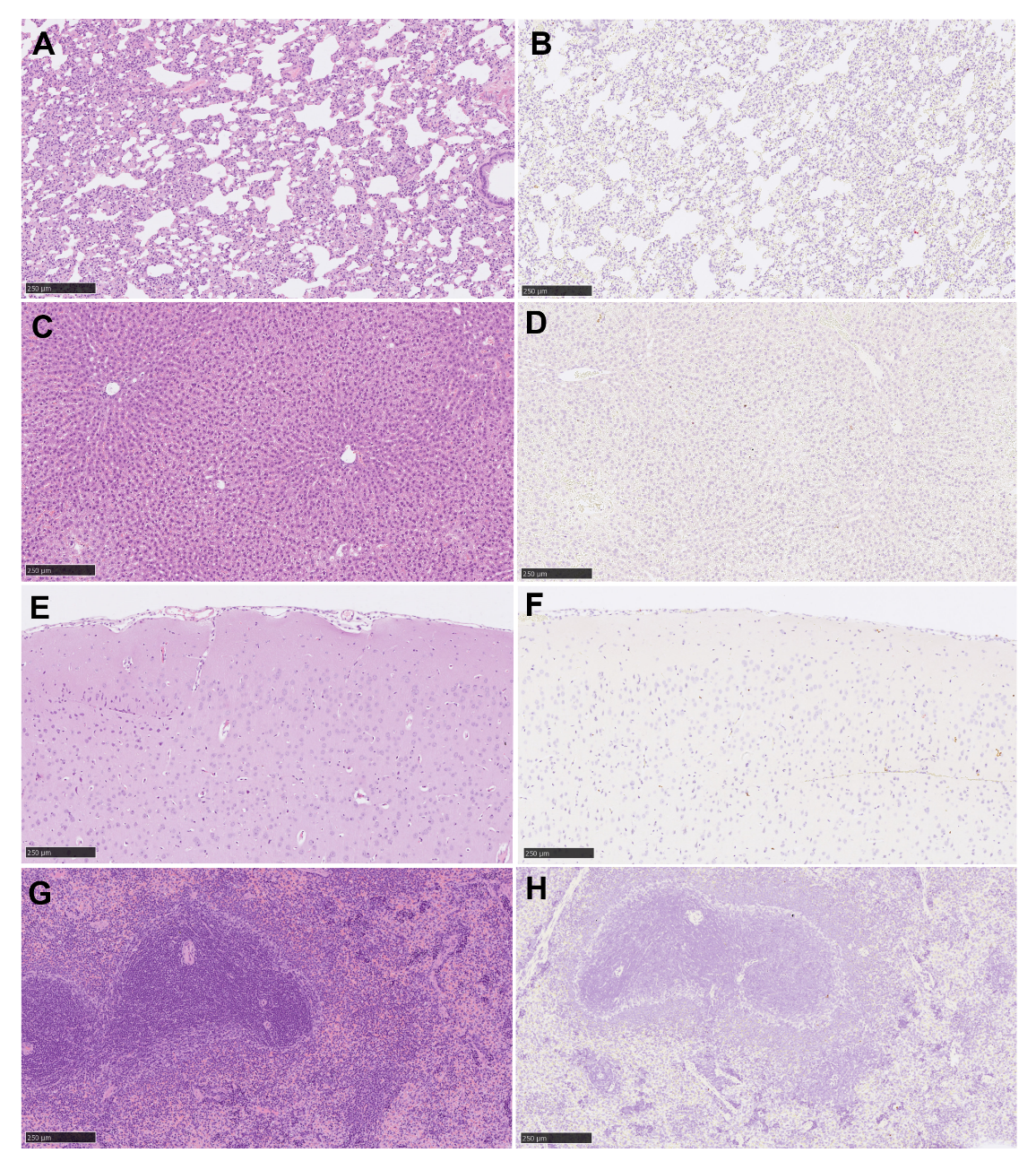


**Supplementary figure 2. Representative histology (H&E) and *in-situ* hybridization (ISH) RNAscope technique in mock group (group 7). (a)** Lung section without histopathological changes. **(b)** Lung section negative against NiV ISH-RNAscope. **(c)** Liver section without histopathological changes. **(d)** Liver section negative against NiV ISH-RNAscope. **(e)** Brain section without histopathological changes. **(f)** Brain section negative against NiV ISH-RNAscope. **(g)** Spleen section without histopathological changes. **(h)** Spleen section negative against NiV ISH-RNAscope. Scale bars = 250 µm.


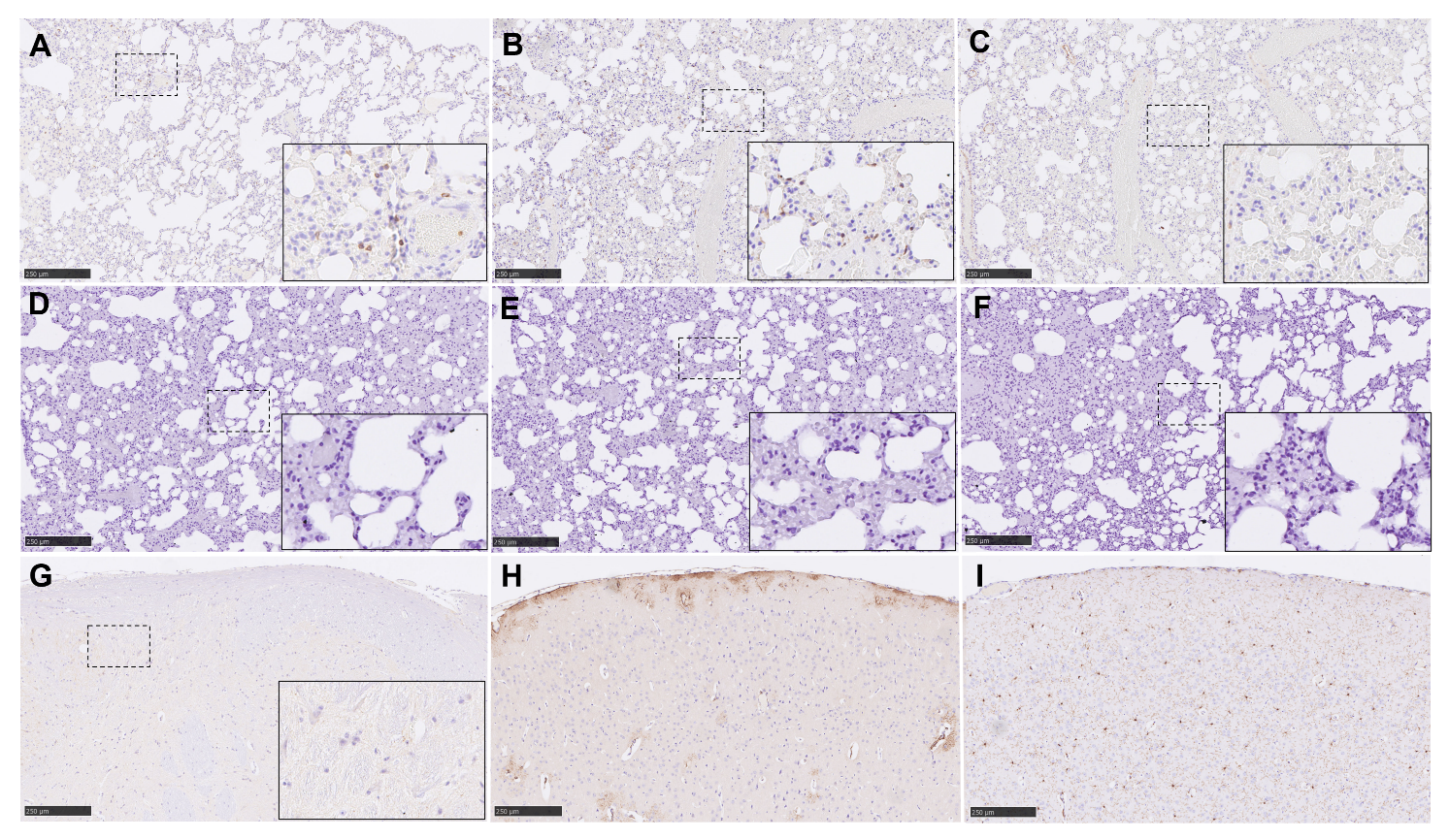


**Supplementary figure 3. Representative immunohistochemistry (IHC) and *in-situ* hybridization (ISH) RNAscope technique in lung and brain from mock group (group 7). (a)** CD3 IHC in lung showing few CD3^+^ T lymphocytes. Inset shows higher magnification. **(b)** Iba1 IHC in lung showing few Iba^+^ macrophages. Inset shows higher magnification. **(c)** NiV N protein IHC in lung showing no positive staining. Inset shows higher magnification. **(d)** IL-6 mRNA ISH-RNAscope in lung showing no positive staining. Inset shows higher magnification. **(e)** TNF mRNA ISH-RNAscope in lung showing no positive staining. Inset shows higher magnification**. (f)** IFNβ1 mRNA ISH-RNAscope in lung showing no positive staining. Inset shows higher magnification. **(g)** CD3 IHC in brain showing no CD3^+^ T lymphocytes. Inset shows higher magnification. **(h)** GFAP IHC in brain showing low number of GFAP^+^ astrocytes. **(i)** Iba1 IHC in brain showing low number of Iba1^+^ microglia cells. Scale bars = 250 µm.

**
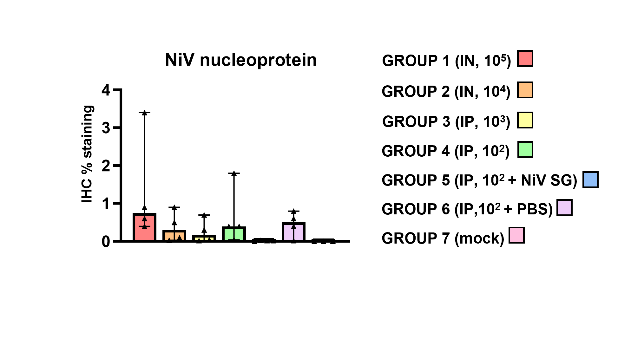
**

**Supplementary figure 4. Quantitative results from NiV nucleoprotein immunohistochemistry (IHC) in lung from different experimental groups**. Data points show values from individual animals (black triangles) with columns and whisker plots denoting median with range. N = 4 animals per experimental group.

**
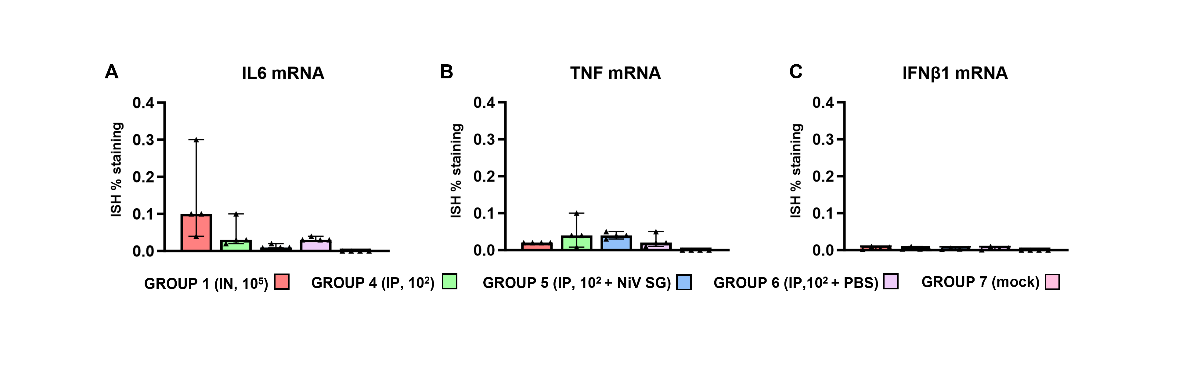
**

**Supplementary figure 5. Quantitative results from IL-6, TNF and IFNβ1 mRNA *in-situ* hybridization (ISH) RNAscope technique in lung from selected experimental groups**. Data points show values from individual animals (black triangles) with columns and whisker plots denoting median with range. N = 4 animals per experimental group.
